# Congenital transmission of *Neospora caninum* in wild ungulates and foxes

**DOI:** 10.3389/fvets.2023.1109986

**Published:** 2023-02-06

**Authors:** Stefania Zanet, Manuela Poncina, Ezio Ferroglio

**Affiliations:** Department Veterinary Sciences, Università degli Studi di Torino, Turin, Italy

**Keywords:** vertical transmission, *Neospora caninum*, roe deer, wild boar, red fox

## Abstract

**Introduction:**

*Neospora caninum* is one of the main causes of abortion in cattle. In wildlife, the occurrence and relevance of vertical infection have not been yet clearly evaluated. The aim of this study was to verify the possibility of vertical transmission of *N. caninum* in three wild species extensively distributed in Europe, namely roe deer *Capreolus capreolus*, wild boar *Sus scrofa* and red fox *Vulpes vulpes*.

**Methods:**

A total of 190 fetuses (72 wild boars, 67 foxes and 51 roe deer) from 61 females were included in the study. All animals, which were either found dead or culled within selective control plans in North-western Italy, were tested, in parallel by PCR on central nervous system, skeletal muscle and kidney.

**Results and discussion:**

The efficiency of vertical transmission in the three target species was of 87.5% (95% CI 69.00–95.66).

## 1. Introduction

*Neospora caninum* is a protozoan parasite, first described in 1988 (1). The main hosts involved in the domestic cycle are dogs (2, 3) and cattle (4) as definitive and intermediate hosts, respectively. *Neospora caninum* is known to have a broad range of wild intermediate hosts. The existence of a sylvatic cycle was first demonstrated in North America, being maintained by coyotes *Canis latrans* (5) and white-tailed deers *Odocoileus virginianus* (6, 7). To date, wild carnivores recognized as competent definitive hosts for *N. caninum* include coyotes (5), dingoes *Canis lupus dingo* (8) and wolves *Canis lupus* (9). *N. caninum* has been detected in a wide spectrum of herbivores, carnivores, rodents and birds which might serve as intermediate hosts (10, 11). Several of the numerically most relevant ungulate species in Europe were found infected with *N. caninum*, including red deer *Cervus elaphus*, roe deer *Capreolus capreolus*, chamois *Rupicapra rupicapra*, alpine ibex *Capra ibex* (12–14), wild boar *Sus scrofa*, fallow deer *Dama dama*, mouflon *Ovis ammon* (15), moose *Alces alces* (16) and bison *Bison bonasus bonasus* (17). The parasite has been demonstrated to infect lagomorphs as brown hare *Lepus europaenus*, wild rabbit *Oryctolagus cuniculus* and eastern cottontail *Sylvilagus floridanus* (18–20). Rodents like the house mouse *Mus musculus*, field mouse *Apodemus sylvaticus* and brown rat *Rattus norvegicus*, are suspected to be an important link between domestic and sylvatic cycles, because of their cosmopolitan diffusion and their role as prey for both domestic and wild canids (10, 21).

*N. caninum* infection is known to produce neuromuscular clinical signs in dogs (22). The parasite is the primary infective cause of abortion in cattle herds worldwide (23, 24), resulting in serious economic losses in farm management (25), which globally amounts to $ 1,298 million (26). Clinical presentation of *N. caninum* infection in cattle includes embryonic resorption, fetal death, abortion, stillbirth or birth of persistently infected animals without clinical signs (22). Vertical transmission is a highly effective route of infection, which in cattle has been reported to occur with a frequency between 81 and 95% (25). Congenital transmission occurs through the passage of tachyzoites across the placenta after ingestion of environmental oocysts during pregnancy or by reactivation of quiescent bradyzoites in chronically infected animals. This is the main route for the maintenance of *N. caninum* in cattle herds (27). The existence of congenital infection has been demonstrated in other species like dog, cat (28), sheep (29), goat (30), non-human primates (31) and mouse (32).

Few case reports dealt with the hypothesis of a vertical transmission of *N. caninum* in captive wild animals. *N. caninum* was diagnosed in a stillborn Eld's deer *Cervus eldi siamensis* (33), in a fallow deer with neurological clinical signs compatible with *N. caninum* infection (34), in two full-termed stillborn twins of lesser kudu *Tragelaphus imberbis* (35) and an Axis deer *Axis axis* died shortly after birth (36). A recent study assessed seroconversion following vertical transmission of *N. caninum* in captive South American deer species (37) while to our knowledge, only one study assessing the possibility of congenital infection in free ranging wild animals, was conducted in North America on white tailed deer (38). Two experimental studies carried out on breeding foxes (*Vulpes vulpes* and *Alopex lagopus*) identified *N. caninum* in cub's tissues, suggesting the occurrence of vertical transmission in this species (39, 40). No data are available about congenital transmission in the target species. In the study area (Piedmont Region, North-western Italy), *N. caninum* is known to circulate within wildlife in ruminants (12, 13), rodents (21), lagomorphs (20) and in domestic dogs (41), cats (42) and cattle (43). In this context, we reported data from the first study conducted with the specific aim of assessing vertical transmission of *N. caninum* in roe deer, wild boar and fox. These three species were chosen due to their high abundance, wide geographical range, and different feeding strategies that make them a good model of the parasite's life cycle in wild animals.

## 2. Materials and methods

### 2.1. Study area and sample collection

**Figure 1:**
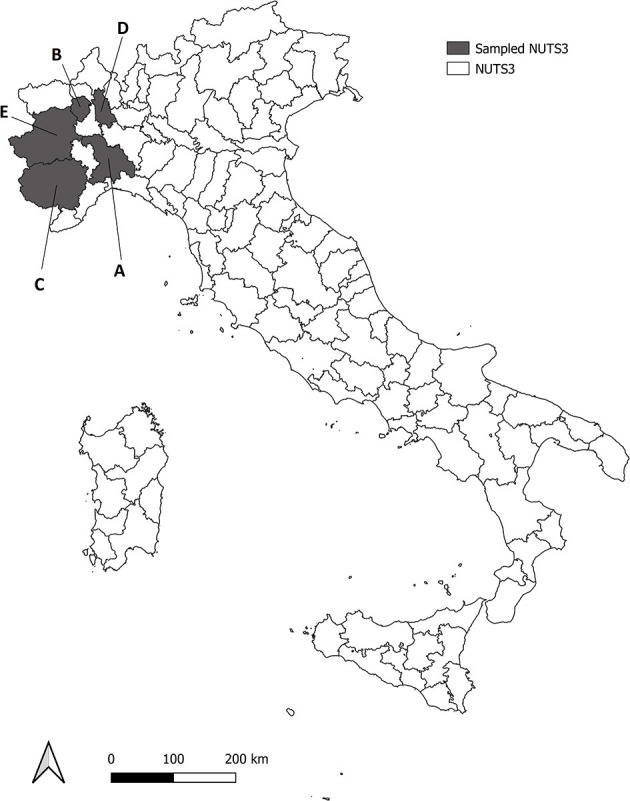
Roe deer, wild boar and red fox analyzed were sampled from five Provinces (NUTS3) in North western Italy, in gray in the map (NUTS3–Nomenclature of Territorial Units for Statistics, level 3). Sampled NUTS3 are Alessandria (area A), Biella (area B), Cuneo (area C), Novara (area D) and Torino (area E).

All the animals included in the study came from five different provinces of North Western Italy (NUTS3–Nomenclature of Territorial Units for Statistics, level 3), namely Alessandria (area A), Biella (area B), Cuneo (area C), Novara (area D) and Torino (area E) (Figure 1), and were taken for necropsy, to the Dept. of Veterinary Sciences (University of Turin) over a period of three consecutive years, if found dead or killed for nationally-authorized selective plans of demographic control. A total of 190 fetuses (*n* = 72 wild boar *Sus scrofa, n* = 67 fox *Vulpes vulpes* and *n* = 51 roe deer *Capreolus capreolus*) were sampled from 61 pregnant females (*n* = 16 wild boar *S. scrofa, n* = 17 red fox *V. vulpes, n* = 28 roe deer *C. capreolus*) (Table 1). Adult females and fetuses were dissected under clean laboratory conditions with sterile scalpels, in order to minimize the risk of cross contamination. After full necroscopic examination, a portion of central nervous system (CNS–*medulla oblongata*), skeletal muscle (*quadriceps femoris*) and kidney of both pregnant females and fetuses were collected and stored at −20°C until further processing. These three tissues were chosen because they were reported to be the most frequently infected in bovine fetuses (43). Samples were immediately frozen and stored in single vials at −20°C until DNA extraction and PCR amplification. Data about origin, weight and sex of the fetuses (in case the stage development allowed sexing procedure) were taken for each animal, and an identification number was assigned to allow an univocal matching between each mother and her fetuses.

**Table 1:** Prevalence of *Neospora caninum* infection in adult females of roe deer, wild boar and red fox.

**Species**		**Roe deer**		**Wild boar**		**Red fox**		**Total**	
Sampled n.		28		16		17		61	
Overall P (CI95%) [n positive]		42.86% (26.51–60.93) [12]		37.50% (18.48–61.36) [6]		35.29% (17.31–58.70) [6]		39.34% (95%CI 28.07–51.88) [24]	
P (CI95%) in Muscle		29.19% (14.91–49.17)		38.46% (17.71–64.48)		31.25% (14.16–55.60)		32.08 % (21.09–45.48)	
P (CI95%) in CNS		25% (11.19–46.87)		8.33% (1.49–35.39)		7.14 (1.27–31.47)		13.73 % (6.81–25.72)	
P (CI95%) in Kidney		23.08% (11.03–42.05)		6.25% (1.11–28.33)		6.25% (1.11–28.33)		13.79 % (7.16–24.93)	
P (CI95%) in sampled NUTS3	A	44.44% (18.88–73.33)		71.43% (35.89–91.78)		-		56.25% (33.18–76.90)	
	B	57.14% (25.05–84.18)		-		0% (0.00–79.35)		50.00% (21.52–78.48)	
	C	42.86% (15.82–74.95)		0% (0.00–79.35)		25% (7.15–59.07)		31.25% (14.16–55.60)	
	D	50% (9.45–90.55)		100% (20.65–100)		50% (18.76–81.24)		55.56% (26.67–81.12)	
	E	0% (0.00–56.15)		0% (0.00–35.43)		50% (9.45–90.55)		8.33% (1.49–35.39)	
Mean age in months (sd)		Neg	Pos	Neg	Pos	Neg	Pos	Neg	Pos
		48.75 (20.30)	44.00 (18.68)	19.9 (9.97)	17.83 (9.50)	21.09 (6.47)	24.0 (0)	32.73 (20.23)	32.45 (18.18)

The number (n.) of individuals of each species and is reported together with the prevalence (P) and Confidence Intervals at 95% (CI95%) for each of the tested tissues (skeletal muscle, central nervous system–CNS and kidney) and geographical area of origin (NUTS3). The mean age (sd, standard deviation) of positive and negative females is expressed in months of age.

### 2.2. PCR analysis

Total genomic DNA was extracted from 25 mg of tissue using GenElute Mammalian genomic MiniPrep Kit (Sigma–Aldrich, St. Louis, MO, USA) according to manufacturer's instructions. A specific 337 bp fragment of the NC5 region of *N. caninum* was amplified using primers Np6-plus (5′-CTCGCCAGTCAACCTACGTCTTCT-3′) and Np21-plus (5′-CCCAGTGCGTCCAATCCTGTAAC-3′), modified from (44), according to (20). Negative controls (*Toxoplasma gondii* DNA and distilled water) were included in each step (extraction, amplification and electrophoresis run), while positive controls (DNA extracted from cultured *N. caninum* NC1 tachyzoites) were included in amplification and electrophoresis run. Positive samples (one amplicon for each mother and litter) were purified using the commercial kit Nucleospin Extract II kit (Macherey-Nagel, Düren, Germany) and sequenced on both strands (MacrogenEurope, The Netherlands) to confirm PCR results. The resulting sequences were compared to those already available in GenBank.

### 2.3. Statistical analysis

Data were analyzed with R-3.4.4 (45). The vertical transmission parameter was calculated as the proportion of PCR positive females that produced PCR positive offspring (at least 1 positive fetus per litter). Uncorrected Chi-square test and Odds ratio were used to determine the association between maternal and fetal infection status. Potential risk factors were analyzed by Generalized Linear Model (GLM), in order to evaluate their effect on the outcome of PCR analysis. Risk factors evaluated were: year of sampling, geographical origin, species, weight (as proxy of gestational stage normalized for each species), tissue tested, sex, PCR positivity of the mother and age of the mother at death. The model with the lower AIC (Akaike Information Criterion) was selected (46). Tests were considered significant when *p*-value (p) was equal or lower than 0.05. In statistical analysis, animals were considered positives if *N. caninum* DNA was found at least in one of the three tissues tested. Cochran Q Test and *post-hoc* McNemar's test were used to evaluate which of the tissue tested (CNS, kidney or skeletal muscle) allowed for higher sensitivity in *N. caninum* identification. Assessment of inter-rater reliability (IRR) to assess PCR results consistency among the three tested tissues was calculated using Light's Kappa (*k*) for multiple raters (47).

## 3. Results

### 3.1. Pregnant females

The overall prevalence of infection in the adult pregnant females was P=39.34% (95%CI 28.07–51.88). *N. caninum* was detected with comparable prevalence among the three species (Table 1). Geographical origin and age (age in months of positive females mea*n* = 32.45, sd = 18.18 *vs*. age of negative females mea*n* = 32.73, *sd* = 20.23) were not significantly associated with PCR results (p>0.05). In the three tested species, skeletal muscle provided the highest sensitivity compared to CNS (χ^2^ = 4.05; *p* < 0.05) and kidney (χ^2^ = 5.79; *p* < 0.05).

### 3.2. Fetuses

The vertical transmission rate of *N. caninum* from PCR positive females was 87.5% (95% CI 69.00–95.66) ranging from 83.33% in roe deer and wild boar, to 100% in red fox (Table 2). In multi-fetus litters (number of fetuses mi*n* = 2, max = 8, sd = 1.68) of infected females, *N. caninum* DNA was detected on average, in 80.00% of the fetuses of the litter (CI95% 0.00–100%). The best performing GLM with *N. caninum* infection in fetuses as independent variable, included two dependent factors, namely fetus weight and PCR status of the mother. Normalized fetus weight resulted as a significant risk factor and likelihood of congenital infection increased with fetus body weight and thus with the gestational stage. In roe deer and wild boar positive fetuses had a significantly higher normalized weight (*p* = 0.0242 and *p* = 0.0002 respectively) compared to negative ones. Congenital infection was strongly associated with positivity of the mother to *N. caninum* (χ^2^ = 47.88, *p* = 0.000; OR = 11.98; CI95% 5.39–28.41%). The association between fetal and maternal infection in the three species is reported in Table 2. No significant correlations were found with the other variables considered (species, year of sampling, origin, sex, mother age) (Table 3). Prevalence of infection in the different tissues ranged from 10.58% (CI95% 6.96–15.78%) in kidneys, to 11.58% (CI95% 7.77–16.91%) in CNS, and 18.09% in skeletal muscle (CI95% 13.24–24.21%). Mc Nemar's test evidenced a significant difference in diagnostic sensitivity between muscle and the other tissues (skeletal muscle-kidney: χ^2^=7.53, *p* = 0.0061; skeletal muscle-CNS χ^2^ = 4.00, *p* = 0.0455) and Light's K underlined a minimal IRR between the three tissues analyzed (*k* = 0.26).

**Table 2:** Association between fetal and maternal positivity to *N. caninum* by PCR in the three species roe deer, wild boar, and red fox.

		**Fetal infection status**		**Efficiency of vertical infection (95% CI)**	**Chi-square (*p*-value)**	**Odds ratio (95% CI)**
**Maternal infection status**	**Roe deer**	Neg	Pos			
	Neg Pos	25	5	83.33% (55.20–95.30)	7.32 (0.007)	5.5 (1.52–19.91)
		10	11			
	**Wild boar**					
	Neg Pos	43	1	83.33% (43.65–96.99)	31.17 (0.000)	66.45 (7.95–555.18)
		11	17			
	**Red fox**					
	Neg Pos	39	6	100% (60.97–100.00)	15.23 (0.000)	9.39 (2.80–31.45)
		9	13			

**Table 3:** Prevalence of *Neospora caninum* infection in fetuses of roe deer, wild boar, and red fox.

**Species**		**Roe deer**		**Wild boar**		**Red fox**		**Total**	
Sampled n.		51		72		67		190	
Overall P (CI95%) [n positive]		31.37% (20.33–45.03) [16]		25.00% (16.44–36.09) [18]		28.36% (18.97–40.09) [19]		[53]	
P (CI95%) in Muscle		25.49% (15.55–38.87)		13.89% (7.72–23.71)		16.42% (9.42–27.06)		18.09% (CI95% 13.24–24.21%)	
P (CI95%) in CNS		13.73% (6.81–25.72)		9.72% (4.79–18.74)		11.94% (6.18–21.83)		11.58% (CI95% 7.77–16.91%)	
P (CI95%) in Kidney		15.69% (8.17–28.01)		5.56% (2.18–13.43)		11.94% (6.18–21.83)		10.58% (CI95% 6.96–15.78%)	
Sex	F	31.82% (16.36–52.68)		37.04% (21.53–55.77)		26.09% (12.55–46.47)		31.94% (22.33–43.39)	
	M	29.17% (14.91–49.17)		26.67% (14.18–44.45)		34.62% (19.41–53.78)		30% (21.06–40.77)	
	U	40% (11.76–76.93)		0% (0.00–20.39)		22.22% (9.00–45.21)		15.79% (7.44–30.42)	
P (CI95%) in sampled NUTS3	A	20% (7.05–45.19)		48% (30.03–66.50)		-		37.50% (24.22–52.97)	
	B	35.71% (16.34-−61.24)		-		33.33 (6.15–79.23)		35.29% (17.31–58.70)	
	C	46.15% (23.21–70.86)		0% (0.00–65.76)		25.93% (13.17–44.68)		30.95% (19.07–46.03)	
	D	0% (0.00–48.99)		75.00% (40.93–92.85)		32.14% (17.93–50.66)		37.50% (24.22–52.97)	
	E	20% (3.62–62.45)		0% (0.00–9.41)		22.22% (6.32–54.74)		5.88% (2.02–15.92)	
Mean weight gr(sd)		Neg	Pos	Neg	Pos	Neg	Pos	Neg	Pos
		409.44 (468.46)	830.36 (690.24)	245.60 (253.93)	513.78 (239.39)	37.66 (34.91)	40.42 (22.81)	213.17 (316.95)	424.33 (498.59)

The number (n.) of individuals of each species and is reported together with the prevalence (P) and Confidence Intervals at 95% (CI95%) for each of the tested tissues (skeletal muscle, central nervous system–CNS and kidney), area of origin (NUTS3) and sex (F, female; M, male; U, undetermined). The mean weight (and standard deviation, sd) of positive and negative fetuses is expressed in grams.

### 3.3. Sequencing

A total of 45 positive samples (each of 24 positive pregnant females and one isolate for each of the 21 positive litters) were sequenced and deposited in GenBank under accession numbers MT346029 to MT346031. Minor differences (max 3 bp) were detected among the isolates, and in all cases isolates from fetuses of each litter were 100% identical to the isolate detected in their respective mothers.

Analysis of the NC5 DNA sequences showed a maximum identity ranging from 98 to 100% (query coverage of 100%), with *N. caninum* isolates from rodents (GenBank accession numbers: EF202079–EF202082) and cattle (GenBank accession numbers: KP715561, KP715562) from the same study area.

## 4. Discussion

To the best of our knowledge, this study evidenced for the first time the possibility of congenital transmission of *N. caninum* in roe deer, wild boar and fox, also assessing vertical transmission on a large number of free-ranging individuals. Indeed, previous evidence of congenital infection in free-ranging wildlife was given only in the white-tailed deer (*O. virginianus*) (38). Transplacental transmission in captive animals was hypothesized by serological evidence (37) or when stillbirth or perinatal neurological signs were indicative of *N. caninum* infection (33–36). *N caninum* DNA was detected in fetal tissues of all the three species considered (wild boar, roe deer, and red fox). At necropsy, none of the fetuses showed any alteration possibly attributable to *N. caninum* infection. PCR positivity for *N. caninum* of the mother was strongly associated with fetus positivity in all three species (OR = 11.98). In wild boar the recorded OR is 66.45 (95%CI 7.95–555.18), while in Roe deer and Fox the OR values are 5.5 (95% CI 1.52–19.91) and 9.39 (95%CI 2.80–31.45) respectively. The recorded efficiency of vertical infection is high in all three target species and falls within the range previously reported for cattle (25). DNA sequencing showed 100% identity between fetal and maternal isolates of *N. caninum*. Even if skeletal muscle showed a significant higher sensitivity than the other tissues, considered the minimal IRR among tissues tested, multiple tissues should always be analyzed in parallel in order to avoid underestimation of parasite's prevalence in fetuses. Fetal weight was considered indicative of gestational age and significantly related to *N. caninum* infection in roe deer and wild boar as previously reported in cattle (22, 48). Pregnancy-induced immunomodulation with reduced cell-mediated response and INFγ production, is a major responsible of parasite's replication control, and allows transplacental transmission of tachyzoites in chronically as well as in newly infected subjects (49–51). Vertical transmission had already been hypothesized in roe deer because of the lack of correlation between seropositivity and age of the tested subjects (12). The authors suggested that the high seroprevalences in animals of all ages, including yearlings, is the result of a concomitant occurrence of both vertical and horizontal transmission. Horizontal transmission of *N. caninum* occurs trough the ingestion of oocyst spread with feces of definitive hosts or trough consumption of preys infected with tissue cysts. Remarkably, horizontal transmission connects the domestic and sylvatic cycles of *N. caninum*. In the study area the main hosts involved in the domestic cycle are cattle and rural shepherd dogs (41). These latter are considered the primary connection with the sylvatic cycle, providing both contamination of pastures with oocysts and by occasionally feeding on wild ungulates. Moreover, the role of wolves in *N. caninum* transmission in the study area is possibly evolving since presence and density of *C. lupus* has been increasing steadily in the past decade and roe deer and wild boars are two of its main preys (52). *N. caninum* isolates, sequenced in this study, are identical to *N. caninum* isolates of rodents and cattle from the same area (21). This finding further corroborates the hypothesis that the sylvatic cycle is maintained by vertical transmission and amplified by the presence of wild predators, acting as definitive hosts. Moreover, this sylvatic cycle seems correlated with the domestic one, not just by domestic dogs, but also by rodents (21). This finding further supports the hypothesis that these species could act as a link between domestic and wild cycle, due to their wide territorial distribution and their role as prey for various domestic and wild species.

The results obtained in this study assessed, for the first time, the possibility of transplacental transmission in roe deer, wild boar and red fox. The role of vertical transmission in establishing and maintaining *N. caninum'*s sylvatic cycle must be confirmed by demonstrating that transplacental infection can lead to the onset of persistent infection in offspring. The high prevalence, found in the wild species here analyzed, underlines their role as intermediate host in the parasite life cycle in an extensive geographical area, characterized by a variety of wild-domestic interfaces. The assessment of the vertical transmission and of an existing connection between wild and domestic parasitic cycles, leads to the need of reconsidering management measures and epidemiological surveillance, with the aim of controlling transmission between wild and domestic animals. Further research is also needed to quantify the relevance of wild carnivores as definitive hosts especially in light of current increasing population trends and geographical expansion of competent hosts (*Canis lupus*) (9).

## Data availability statement

The original contributions presented in the study are included in the article/supplementary material, further inquiries can be directed to the corresponding author/s.

## Ethics statement

Ethical review and approval was not required for the study on animals in accordance with the local legislation and institutional requirements.

## Author contributions

SZ: investigation, data curation, formal analysis, and writing final version of manuscript. MP: investigation, writing—original draft, and review and editing. EF: conceptualization, funding acquisition, and writing—review and editing. All authors contributed to the article and approved the submitted version.
